# Impact of non-drug therapies on asthma control: A systematic review of the literature

**DOI:** 10.1080/13814788.2019.1574742

**Published:** 2019-03-08

**Authors:** Matthieu Schuers, Anthony Chapron, Hugo Guihard, Tiphanie Bouchez, David Darmon

**Affiliations:** a Department of General Medicine, Rouen University, Rouen, France;; b Department of General Medicine, Rennes University, Rennes, France;; c Department of General Medicine, Nice University, Nice, France

**Keywords:** Asthma, systematic review, chronic disease, therapy

## Abstract

**Background:** Despite growing access to effective therapies, asthma control still needs improvement. Many non-drug factors, such as allergens, air pollutants and stress also affect asthma control and patient quality of life, but an overview of the effectiveness of non-drug interventions on asthma control was lacking.

**Objectives:** To identify non-drug interventions likely to improve asthma control.

**Methods:** A systematic review of the available literature in Medline and the Cochrane Library was conducted in March 2017, without any time limit. Initial searching identified 884 potentially relevant clinical trial reports, literature reviews and meta-analyses, which were screened for inclusion using criteria of quality, relevance, and reporting outcomes based on asthma control.

**Results:** Eighty-two publications met the inclusion criteria. In general, the quality of the studies was low. Patient education programmes (22 studies) significantly improved asthma control. Multifaceted interventions (10 studies), which combined patient education programmes with decreasing exposure to indoor allergens and pollutants, significantly improved asthma control based on clinically relevant outcomes. Renovating homes to reduce exposure to allergens and indoor pollutants improved control (two studies). Air filtration systems (five studies) were effective, especially in children exposed to second-hand smoke. Most measures attempting to reduce exposure to dust mites were ineffective (five studies). Dietary interventions (eight studies) were ineffective. Promoting physical activity (five studies) tended to yield positive results, but the results did not attain significance.

**Conclusion:** Twenty-six interventions were effective in asthma control. Simultaneously combining several action plans, each focusing on different aspects of asthma management, seems most likely to be effective.

KEY MESSAGES

- Therapeutic patient education programmes significantly improve disease control.
- Multifaceted interventions, combining patient education programmes with measures to decrease exposure to indoor allergens and pollutants, significantly improved disease control.
- These results call for a stronger emphasis on patient-focused care in asthma, in particular on their information needs and self-management skills.

## Introduction

About 300 million people have asthma worldwide, including 30 million in Europe [1,2]. Asthma mortality has decreased in recent years, most likely because of new treatments and the spread of clinical guidelines but there is still room for improvement [3,4]. Patients with asthma often suffer from comorbidities and these comorbid diseases may hinder asthma control [5–8].

The publication of the Global Initiative for Asthma (GINA) recommendations for asthma in 2004 marked a shift from the concept of severity to that of control [9]. Control of asthma is evaluated based on disease activity in the last four weeks, assessed by the frequency of respiratory symptoms and their impact on daily living.

The effectiveness of drug treatments for asthma is well-recognized, with inhaled corticosteroids the cornerstone of treatment [9]. But many other factors are also associated with asthma control, including allergens, air pollutants, viral infections, foods, drugs—non-steroidal anti-inflammatory drugs (NSAIDS), beta-blockers—obesity and emotional stress [9,10]. Addressing them could help to improve asthma control and patient quality of life.

Studies that attempt to measure the effectiveness of interventions aimed at correcting these factors are more difficult to perform than drug trials, may suffer from contamination bias, and be of doubtful generalizability. However, as a chronic disease, asthma calls for comprehensive care. The goal of this review is to identify and summarize the published evidence concerning non-drug interventions that aim to improve asthma control in adults and children.

## Method

A systematic review of the available literature was conducted in March 2017. There were no time limits.

### Information sources

The Medline database (PubMed) and the Cochrane Library were used to identify relevant published articles.

### Search strategy

We searched PubMed to find all articles indexed using the MeSH terms: ‘asthma,’ ‘risk factors’ and ‘prevention and control,’ then limited the search to include only those articles classified as a ‘clinical trial,’ ‘review,’ ‘systematic review’ or ‘meta-analysis.’ In the Cochrane Library, the term ‘asthma’ was sought in titles, abstracts and keywords. When an original intervention study had already been aggregated into a review, we excluded the original study.

### Inclusion criteria

Articles were judged potentially relevant to our review if they:

- studied a population of adults and children with asthma. Participants could be on medication, as long as the medication were not part of the intervention;
- were clinical trials, reviews or meta-analyses of non-drug interventions for asthma;
- studied interventions of non-drug therapies, though we included vaccination studies as our goal was to build a comprehensive overview of all the available preventive strategies;
- reported outcomes based on asthma control, including at least one defined by GINA—day or night symptoms, physical activity, exacerbations, absence from work or school, use of short-acting (rescue) β_2_-agonists, forced expiratory volume in one second (FEV_1_) or peak expiratory flow (PEF), and circadian variation of the PEF. Each study’s primary outcome measure(s) was used to judge the effectiveness of the intervention. The lack of standard outcome measures across the different studies meant that we were unable to define clinically relevant improvements in each outcome in advance;
- were written in English or French.

We excluded reports of interventions which only targeted exacerbations, or which were primarily concerned with the effectiveness of one or more drugs.

### Selection process

The list of articles identified in the database was established, and duplicate entries were eliminated. Each article was analysed for inclusion by two independent investigators. Disagreements were resolved by consensus.

### Data extraction and analysis

**Figure 1.**
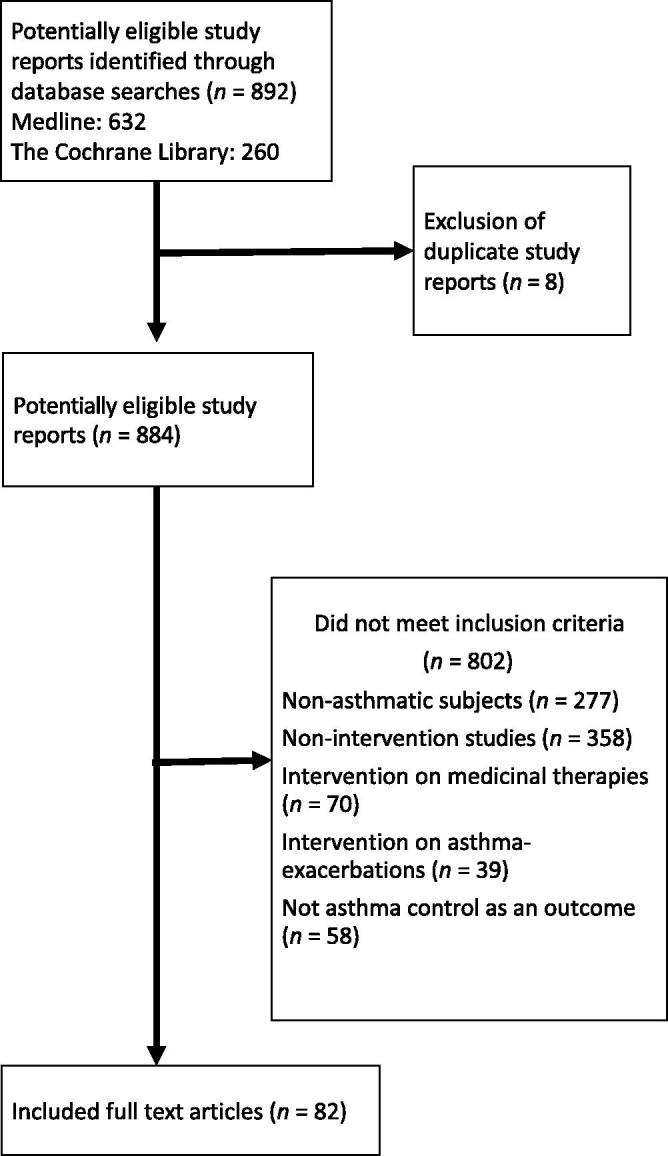
Study selection flow chart.

Eight hundred and ninety-two references were identified. Eight duplicate study reports were excluded. Eighty-two references were included. The selection process is summarized in Figure 1. The name of the first author, year of publication, country where the study was conducted, study design, number and age of participants, type and description of intervention, primary outcomes and estimated effect size with corresponding 95% confidence intervals were extracted and recorded in an Excel spreadsheet.

## Results

The original articles reported studies conducted mainly in the US, Canada, Australia and Northern Europe, which included both children and adults. Most were performed in primary care.

### Indoor environment

A meta-analysis of 23 randomized controlled trials (RCTs) focused on physical methods (such as air aspiration systems) or chemical methods (miticides) to reduce exposure to dust mite allergens in the homes of adults or children with asthma who were sensitive to acarids (Table 1), [11–27]. These interventions were found not to improve asthma control when used in an isolated manner^11^. Renovating homes affected by dampness or mould improved adults’ breathing symptoms and reduced emergency treatment delivered to children [13]. Workplace interventions attempting to reduce or eliminate exposure to airborne pathogens significantly improved symptoms [15].

**Table 1.** Description of selected studies evaluating indoor living environment interventions to improve asthma control (*n* = 17).

Reference	Intervention	Population	Effectiveness on primary outcome 95%CI	Type of study
[11]	Efficient heating device	Children (*n* = 409)	Less absenteeism from school AD: –1.80 day (–3.10, –0.11)	RCT
[12]	Miticide cleaning supplies	Children allergic to dust mites (*n* = 26)	Reduction of the symptoms (*P <*0.01)	RCT
[13]	Renovating homes with humidity and mould problems	Adults and children (*n* = 6 538)	Reduction of the adults’ symptoms OR: 0.64 (0.55, 0.75)	Meta-analysis
[14]	Building new homes meant to reduce exposure to dust mites and indoor allergens	Children (*n* = 102)	Fewer resorts to unscheduled treatments (%) 41.2 (65.9, 16.5)	Quasi-experimental
[15]	Ending exposure at work	Adults (*n* = 1 447)	Increased probability for not exhibiting any symptoms RR: 21.42 (7.20, 63.77)	Meta-analysis
[16]	Filtration air purifier	Children exposed to passive smoking (*n* = 126)	Increase in the number of symptomless days (*P* = 0.03)	RCT
[17]		Children (*n* = 225)	Fewer resorts to unscheduled treatments (*P* = 0.043)	RCT
[18]		Adults and children (*n* = 216)	Reduction of the symptoms WAD: –0.47 (–0.69, –0.25)	Meta-analysis
[19]		Adults and children (*n* = 40)	No evidence of effectiveness	RCT
[20]		Adults and children (*n* = 57)	No evidence of effectiveness	Syst. Rev.
[21]	Dehumidifier	Adults (*n* = 159)	No evidence of effectiveness	Syst. Rev.
[22]	Ionic air purifier	Adults and children (*n* = 106)	No evidence of effectiveness	Meta-analysis
[23]	Anti-dust mite blankets	Adults (*n* = 1 122)	No evidence of effectiveness	RCT
[24]	Feather pillows and quilts	Children allergic to dust mites (*n* = 197)	No evidence of effectiveness	RCT
[25]	Anti-allergic pillows and quilts	Children (*n* = 104)	No evidence of effectiveness	RCT
[26]	Chemical and physical methods to reduce exposure to dust mites	Adults and children (*n* = 686)	No evidence of effectiveness	Meta-analysis
[27]	Video- and telephone-based intervention to reduce exposure to dust mites and indoor allergens	Adults (*n* = 300)	No evidence of effectiveness	RCT

95%CI: 95% confidence interval; AD: average difference; OR: odds ratio; RCT: randomized control trial; RR: relative risk; Syst. Rev.: systemic review of the literature; WAD: weighted average difference.

The use of air purifiers in living rooms and children’s rooms was assessed in several studies [16–22]. A meta-analysis of air filtration reported an association with fewer symptoms, but none of the trials had employed validated scales to measure outcomes [18]. Two RCTs showed a reduction of symptoms in inner-city children [16] or on the use of unscheduled asthma visits in children exposed to second-hand smoke at home [17]. Systems using ionizers or dehumidifiers were not effective [21,22]. Adapting bedding as the sole measure to reduce exposure to dust mite allergens showed no positive effect on asthma control [23–25].

### Diet and exercise

Fourteen literature reviews and two meta-analyses focused on diet or physical activity (Table 2), [28–43]. Diets enriched with vitamin C, marine fatty acids, or selenium did not have any effect on asthma control [28–30]; neither did restricting sodium or eliminating monosodium glutamate [31,32]. Caffeine improved the peak expiratory flow (PEF), but only for four hours after consumption [33].

**Table 2.** Description of selected studies evaluating dietary and physical activity interventions to improve asthma control (*n* = 15).

	Intervention	Population	Effectiveness on primary outcome 95%CI	Type of study
[33]	Caffeine intake	Adults (*n* = 75)	PEF improvement (%) 5.47 (1.43, 9.52) (Not clinically relevant)	Meta-analysis
[36]	Low-calorie diet	Adults (*n* = 38)	No evidence of effectiveness	Syst. Rev.
[31]	Low-salt diet	Adults (*n* = 381)	No evidence of effectiveness	Syst. Rev.
[32]	Low monosodium glutamate diet	Adults (*n* = 24)	No evidence of effectiveness	Syst. Rev.
[29]	Marine fatty acid-enriched diet	Adults and children (*n* = 187)	No evidence of effectiveness	Syst. Rev.
[30]	Selenium-enriched diet	Adults and children (*n* = 24)	No evidence of effectiveness	Syst. Rev.
[28]	Vitamin C supplementation	Adults and children (*n* = 330)	No evidence of effectiveness	Syst. Rev.
[37]		Adults and children (*n* = 419)	No evidence of effectiveness	Syst. Rev.
[38]	Vitamin C and E supplementation	Adults and children (*n* = 214)	No evidence of effectiveness	Syst. Rev.
[34]	Physical activity	Adults and children (*n* = 695)	No evidence of effectiveness	Syst. Rev.
[39]		Adults (*n* = 772)	No evidence of effectiveness	Syst. Rev.
[40]	Breathing exercises	Adults (*n* = 906)	No evidence of effectiveness	Syst. Rev.
[41]	Inspiratory muscles training	Adults (*n* = 113)	No evidence of effectiveness	Syst. Rev.
[35]	Swimming	Children and adolescents (*n* = 262)	No evidence of effectiveness	Meta-analysis
[42]	Water based activity	Adults (*n* = 136)	No evidence of effectiveness	Syst. Rev.

95%CI: 95% confidence interval; RCT: randomized control trial; Syst. Rev.: systemic review of the literature.

Physical activity did not cause any side effects and did not exacerbate symptoms. Although the evidence lacked strength, the authors suggested that promoting physical activity improved quality of life [34,35]. One study of 38 patients included in a 2002 Cochrane review reported that low-calorie diets had beneficial effects on asthma control; however, the review authors considered that the evidence was inconclusive [36]. A Cochrane review of weight loss interventions in patients with asthma retrieved a controlled study which showed a short-term reduction in doses of rescue medication in the treatment group. Weight loss was associated with a statistically significant improvement in FEV_1_ and FVC in one study, but this was considered clinically unimportant; there was no improvement in PEF. No data were reported on healthcare utilization and adverse effects. The reviewers concluded that poor study methodology meant that any positive effect of obesity treatment on asthma control was uncertain [43].

### Vaccinations

A review of the use of the pneumococcal vaccine in patients with asthma found only one study, of 80 children aged 2 to 6 years. The authors considered that there was insufficient evidence to recommend pneumococcal vaccine for patients with asthma [44].

Eighteen articles were included in a recent Cochrane review of flu vaccination for patients with asthma. Only two high-quality articles assessed the impact of the vaccine on the number of exacerbations, but their results did not demonstrate any beneficial effects of flu vaccination on patients with asthma [45].

### Alternative or additional therapies

Several Cochrane reviews have studied alternative therapies, such as acupuncture, homeopathy or herbal medicine [46–48]. There was no evidence to support the use of these therapies in treating asthma. A review of speleotherapy (a method based on giving treatment in an underground environment) demonstrated non-significant improvement on the PEF [49].

### Physiotherapy

Two Cochrane reviews studied physiotherapy. The first review, of breathing exercises in patients of all ages, included seven articles. The heterogeneous nature of the interventions and effectiveness criteria precluded the authors from drawing any robust conclusions for practice [50]. Two articles about manual therapies, such as osteopathy, were included in another review, and found no evidence of effectiveness [51].

### Psychological treatment

Four meta-analyses focused on psychological treatment. The use of relaxation techniques was found to decrease consumption of drugs ‘on demand’ significantly and increased the PEF by 31.73 L/min (*P* < 0.0001). Cognitive behavioural therapy significantly improved the quality of life, as measured by the asthma quality of life questionnaire. Methodological limitations prevented the authors from drawing definite conclusions about the effect these interventions could have on asthma control [52].

A second review devoted to psychological treatment of children with asthma yielded similar conclusions [53], though children undergoing family therapy and receiving pharmacological treatment were less likely to limit their activities and reported fewer days with dyspnoea and wheezing [54].

The use of written emotional disclosure did not improve asthma control [55].

### Therapeutic education

Therapeutic patient education (TPE) aims to help patients acquire and maintain the necessary skills to self-manage their chronic disease Table 3, [56–78]. Four Cochrane reviews reported a positive impact of TPE on asthma control. TPE decreased night-time symptoms, the number of days of restricted activity, and increased quality of life. It reduced the numbers of days lost at work or school, the use of ambulances, and emergency department (ED) visits, though it did not significantly reduce ED re-presentations. While the trend in effect favours educational interventions, the pooled results were not statistically significant. TPE seemed to reach higher effectiveness in uncontrolled asthma, especially in children [57–60].

**Table 3.** Description of selected studies evaluating therapeutic patient education interventions to improve asthma control (*n* = 22).

Reference	Intervention	Population	Effectiveness on primary outcome 95%CI	Type of study
[58]	Therapeutic patient education (TPE) programme	Adults (*n* = 6090)	Smaller risk of hospitalization RR: 0.64 (0.50, 0.82) Fewer emergency room visits RR: 0.82 (0.73, 0.94) Fewer unscheduled GP visits RR: 0.68 (0.56, 0.81)	Meta-analysis
[59]		Children (*n* = 3706)	Reduced absenteeism from school SMD: –0.14 (–0.23, –0.04) Fewer days with restricted activity SMD: –0.29 (–0.49, –0.08) Fewer emergency room visits SMD: –0.21 (–0.33, –0.09)	Meta-analysis
[65]		Adults (*n* = 81746)	Improved quality of life SMD: 0.22 (0.08, 0.37)	Meta-analysis
[66]	TPE programme	Children (*n* = 53)	Fewer emergency room visits –79% (*P* < 0.0001)	Before-after study
[60]	TPE programme after an admission to an emergency department^a^	Adults (*n* = 2157)	Smaller risk of hospitalization RR: 0.5 (0.27, 0.91)	Meta-analysis
[57]		Children (*n* = 7843)	Smaller risk of hospitalization RR: 0.79 (0.69; 0.92)	Meta-analysis
[68]	TPE in a school environment^a^	Adolescents (*n* = 345)	Reduced absenteeism from school RR: 0.63 (0.46, 0.85)	RCT
[69]	Comparison between a structured TPE and limited data	Adults (*n* = 98)	Fewer admissions to an emergency department (*P* = 0.03)	RCT
[72]	TPE based on sending text messages^a^	Adults (*n* = 182)	Reduced clinical score AD: –0.36 (–0.56, –0.17)	Syst. Rev.
[75]	TPE adapted to culture^b^	Children and adults (*n* = 133)	Reduced absenteeism from school –21% (–5%, –36%)	Meta-analysis
[76]		Adults and children (*n* = 617)	Improved quality of life WAD: 0.25 (0.09, 0.41)	Meta-analysis
[77]		Children (*n* = 221)	Smaller risk of hospitalization OR: 0.32 (0.15, 0.72)	RCT
[62]	Use of written action plans (WAPs)	Adults (*n* = 26)	Fewer night-time symptoms (*P* = 0.005)	Before-after study
[63]		Adults (*n* = 2 460)	No evidence of effectiveness	Syst. Rev.
[64]	Comparison between WAPs based on the PEF and WAPs based on the symptoms	Children (*n* = 355)	Fewer emergency treatments administered with symptom-based WAPs RR: 0.73 (0.55, 0.99)	Meta-analysis
[70]		Children and adolescents (*n* = 150)	Fewer emergency treatments administered with PEF-based WAPs (*P* = 0.002)	RCT
[71]		Adults and children (*n* = 149)	No evidence of effectiveness	RCT
[67]	TPE at home^a^	Children (*n* = 2 342)	No evidence of effectiveness	Meta-analysis
[78]	Smartphone and tablet self-management app	Adults (*n* = 408)	No evidence of effectiveness	Syst Rev
[73]	TPE on the Internet^c^	Children (*n* = 438)	No evidence of effectiveness	RCT
[74]	TPE based on solving problems^c^	Adults (*n* = 333)	No evidence of effectiveness	RCT
[61]	TPE based on limited data	Adults (*n* = 906)	No evidence of effectiveness	Meta-analysis

a Comparison with daily treatments.

b Comparison with a standard TPE programme or with daily treatments.

c Comparison with a standard TPE programme.

95%CI: 95% confidence interval; AD: average difference; PEF: peak expiratory flow; RCT: randomized control trial; RR: relative risk; SMD: standardized mean difference; Syst. Rev.: systemic review of the literature; WAD: weighted average difference; WAPs: written action plans.

In another Cochrane review, a restricted health education programme, which included only information related to asthma, its causes, and treatments, did not seem to improve control [61]. The use of written action plans (WAPs) seemed to have a positive impact on night-time symptoms and the number of ED attendances, but the risk of methodological bias (in a before/after study) prompted caution [62]. The result stood in contrast to a Cochrane review that concluded that providing WAPs to adult patients offered no advantages over routine care [63]. Using WAPs with children reduced the number of exacerbations requiring intensive care. WAPs based on symptoms seem preferable to WAPs based on PEF measurement [64].

### Healthcare organization

Four meta-analyses focused on healthcare organization. Nurse-led asthma clinics in primary care settings seem to offer few advantages over standard care [79]. A meta-analysis comparing care delivered by a specialized nurse to that delivered by a GP did not reveal any significant difference in control or quality of life [80].

Pharmacy advice in low- and middle-income countries improved the quality of life (an increase of 0.31 points on a 1 to 5 scale; *P* < 0.001) and decreased GP consultations (*P* = 0.01) [81].

Telemedicine interventions reduced the risk of hospitalization (RR: 0.25; 95%CI: 0.09–0.66), particularly for patients with severe asthma [82].

An RCT comparing nurse-led care in a school setting with routine care found that the intervention decreased night-time symptoms (1.68 nights with symptoms vs. 2.20; *P* = 0.02) and school absence (0.37 days vs. 0.85; *P* = 0.03) [83]. Another RCT of an educational intervention following ED attendance for asthma showed no difference in the number of subsequent ED visits, medication use, or quality of life [84].

### Multifaceted interventions

Several RCTs focused on multifaceted interventions (i.e. those combining several interventions) conducted in primary care in a community context (school, home, local services), or in the ED Table 4, [85–94]. The interventions were heterogeneous, but most included a TPE action plan. Interventions were directed towards children with asthma sensitive to dust mites or exposed to passive smoking, to reduce indoor pollution within patients’ homes [86]. Other measures, such as administering treatment at school, offering telephone follow-up, or delivering patient-centred care regardless of coordination by the GP, were also assessed.

**Table 4.** Description of selected studies evaluating multifaceted interventions to improve asthma control (*n* = 10).

Reference	Intervention	Population	Effectiveness on primary outcome 95%CI	Type of study
[85]	Social workers: - Education care plan - Offer help from other medical-social professionals	Children (*n* = 1 033)	Day-time symptoms reduced –0.55 symptom days (*P* = 0.004)	RCT
[86]	Research assistants’ visits to homes: - Educate - Reduce exposure to tobacco and allergens - Offer follow-up by telephone	Children with atopic asthma (*n* = 937)	Day-time symptoms reduced 3.39 vs 4.20 days (*P* < 0.001)	RCT
[87]	Paediatric Emergency Department: - Educate - Reduce exposure to tobacco and allergens - Organize medical follow-up	Children (*n* = 488)	Fewer unscheduled visits for asthma care RR: 0.60 (0.46; 0.77) More children without limitation in daytime quality of life RR: 1.36 (1.06; 1.73)	RCT
[88]	School education programme involving teachers, health care professionals and city officials	Children (*n* = 66)	Participation in day-to-day activities improved (*P* < 0.01)	RCT
[89]	PAIR-UP intervention: - Prompts for clinician - Practice-level educational support - Practice-level performance feedback	Children (*n* = 638)	More symptom-free days per 2 weeks MD: 0.78 days (0.29, 1.27)	RCT
[90]	School Programme: - Pharmacological treatment administered by a nurse in a school environment - Offer parents a nicotine withdrawal programme	Children (*n* = 530)	More symptom-free days per 2 weeks AD: 0.92 days (0.50, 1.33)	RCT
[91]	Health Visitor: - Educate - Reduce exposure to tobacco and allergens	Children (*n* = 149)	Overall symptoms reduced among children with low severity asthma (*P* = 0.03)	RCT
[92]		Children (*n* = 181)	No evidence of effectiveness	RCT
[93]	Community Health Agents: - Educate - Reduce exposure to tobacco and allergens	Children (*n* = 191)	Fewer hospitalizations 36.5% vs 59.1% (*P* = 0.02)	RCT
[94]		Children (*n* = 274)	No evidence of effectiveness	RCT

95%CI: 95% confidence interval; AD: average difference; AMD: average mean difference; MD: mean difference; RCT: randomized control trial; RR: relative risk.

These multifaceted interventions were not conducted by doctors but were led by social workers or community health workers. Among the 10 studies included, only two did not exhibit any improvement of clinical signs related to control [92, 94]. One study showed a decrease in the number of hospitalizations [93]. Six interventions reduced symptoms and one improved daily activities [85–87, 91].

## Discussion

### Main findings

A total of 82 publications met the inclusion criteria. In general, study methodological quality was low. Out of 68 interventions studied, 26 were effective in asthma control according to the authors’ prospective criteria. Patient education programmes (22 studies) significantly improved asthma control but identifying the most effective type of programme proved difficult. Multifaceted interventions (10 studies), which typically combined therapeutic patient education programmes with decreasing exposure to indoor allergens and pollutants, significantly improved asthma control based on clinically relevant outcomes. Totally or partially renovating homes to reduce exposure to allergens and indoor pollutants improved control (two studies). Air purification systems by filtration (five studies) were effective on asthma control especially in children exposed to second-hand smoke. Most measures attempting to reduce exposure to dust mites were ineffective (five studies). Dietary interventions (eight studies) were ineffective. Physical activity (five studies) had encouraging but insignificant results. Psychological interventions (four studies) and physiotherapy (two studies) were not effective.

### Comparison with existing literature

International guidelines now emphasize the importance of patients being educated to develop the skills to manage their asthma. The GINA components for effective guided asthma self-management include self-monitoring of symptoms and/or peak flow, written asthma action plans and regular review of asthma control, treatment and skills [9]. Our review confirms that therapeutic patient education programmes significantly improve asthma control. However, identifying the most effective therapeutic patient education programmes remains difficult.

Although coordination of care is considered to be part of a GP’s general skill [95], our study shows that effective multifaceted interventions in asthma were generally not conducted by GPs, and that GP-only interventions rarely had a significant impact. Interventions by multi-professional teams seem necessary. In France, some of these multifaceted interventions have materialized in the recent engagement of health advisors in indoor environment.

Most of the measures to reduce exposure to dust mites proved ineffective, but renovating homes, partly or fully, to reduce exposure to allergens and indoor pollution was an effective method to improve control. GINA guidelines also report these data but avoidance strategies are often complicated and expensive [9]. Our study also suggests that air purification using a filter system has a positive impact on asthma control in children exposed to air pollution or second-hand smoke. Further studies employing personal monitoring devices for allergen, pollutant, and microbial exposure may clarify the importance of environmental interventions [96]. There was no evidence to support the use of ionizers. These devices release nitric oxide, which is an asthma trigger [97].

According to GINA, there is a heterogeneous level of evidence regarding isolated measures to reduce outdoor allergens or air pollution [9]. As expected, no strong evidence was found in our study regarding these risk factors but avoiding physical activity in unfavourable environmental conditions seems advisable.

There was no conclusive evidence for specific dietary interventions, and physical activity showed encouraging but non-significant results. However, GINA recommends a healthy diet and regular physical activity for their general health benefits, even if the evidence for one form of physical activity over another remains limited [9].

Respiratory viruses trigger asthma exacerbations [98]. For this reason, GINA logically recommends flu vaccination while acknowledging that it has not proved effective in asthma control [9]. GINA does not recommend the pneumococcal vaccine, and the present review has not identified sufficient evidence to recommend it [9].

Specific interventions included in this review did not address tobacco cessation. Some trials assessing the impact of tobacco cessation on asthma control have been published. The few studies that we identified did not meet our inclusion criteria. In one study participants used medication (oral nicotine) for tobacco cessation and in another, the primary outcome was the change in reported smoking habits after the intervention. It can be assumed that this intervention has mostly been studied by observational and cohort studies that were not included in our review [99]. Nevertheless, tobacco cessation was a frequent element in the multifaceted interventions we did include and helping smokers to quit must remain a key issue for primary care professionals, especially among patients suffering from chronic respiratory conditions.

### Strengths and limitations

To the best of our knowledge, this is the first attempt to synthesize knowledge about the impact of non-drug therapies on asthma control. All the interventions included used patient-centred clinical criteria for asthma control as defined by GINA.

The main study limitation lies within the Medline and Cochrane focus. We chose to include original studies as well as systematic reviews and meta-analysis, though for each intervention, we did not include any original study that had already been systematically reviewed. The alternative would have been to perform a meta-review, i.e., a review of reviews, to ensure the homogeneity of included material. However, this would have limited our attempt to build a comprehensive overview of the topic, in particular regarding multifaceted interventions.

### Implications for clinical practice, research and policy

Simultaneously combining several action plans, each focusing on different factors of asthma control, seems to be the most effective measure. Involvement of both patients and of healthcare professionals is essential, building effective, thorough and multidimensional care for patients with asthma. An example is the French EPODE programme. This programme is a coordinated, capacity-building approach aimed at reducing childhood obesity through a societal process in which local environments, childhood settings, and family norms are directed and encouraged to facilitate the adoption of healthy lifestyles in children [100]. The EPODE programme has demonstrated a global diminution on overweight and obesity prevalence [101], an efficiency across all socioeconomic levels, and the capacity to decrease health inequities [102]. This programme is derived from Wagner’s model, which is otherwise known as the Chronic Care Model [103]. In the case of asthma, an action plan was designed directly from Wagner’s model intended for a sample of children living in a precarious environment [104]; the results of which corroborate the effectiveness of this model on clinical outcomes [90].

Future recommendations regarding asthma should certainly take the efficacy of multifaceted and multidisciplinary interventions into account as well as comorbidity, which mostly affects patients with chronic diseases.

## Conclusion

Most of the effective asthma control interventions focused either on patient education or a combination of a patient education programme with measures to reduce the exposure to allergens and indoor pollution. Recent studies have shown that these interventions can be successfully adapted into primary care settings, reducing the morbidity of asthma in these populations [105]. Future non-drug intervention studies should acknowledge the necessity of a multifaceted approach, and the engagement of a multidisciplinary team. This review may also serve as a summary of the effectiveness of non-drug therapies for asthma.

## Supplementary Material

Supplemental Material - PRISMA 2009 Checklist

## References

[1] Masoli M, Fabian D, Holt S, et al. The global burden of asthma: executive summary of the GINA Dissemination Committee report. Allergy. 2004;59:469–478. 15080825 10.1111/j.1398-9995.2004.00526.x

[2] To T, Stanojevic S, Moores G. et al. Global asthma prevalence in adults: findings from the cross-sectional world health survey. BMC Public Health. 2012;12:204. 22429515 10.1186/1471-2458-12-204 PMC3353191

[3] Tual S, Godard P, Bousquet J, et al. Diminution de la mortalité par asthme en France. Rev Mal Respir. 2008;25:814–820. 18946406 10.1016/s0761-8425(08)74346-8

[4] Demoly P, Paggiaro P, Plaza V, et al. Prevalence of asthma control among adults in France, Germany, Italy, Spain and the UK. Eur Respir Review. 2009;18:105–112. 10.1183/09059180.00001209 20956130

[5] Soriano JB, Visick GT, Muellerova H, et al. Patterns of comorbidities in newly diagnosed COPD and asthma in primary care. Chest. 2005;128:2099–2107. 16236861 10.1378/chest.128.4.2099

[6] Wijnhoven HAH, Kriegsman DMW, Hesselink AE, et al. The influence of co-morbidity on health-related quality of life in asthma and COPD patients. Respir Med. 2003;97:468–475. 12735662 10.1053/rmed.2002.1463

[7] Lehrer PM, Karavidas MK, LuS-E, et al. Psychological treatment of comorbid asthma and panic disorder: a pilot study. J Anxiety Disord. 2008;22:671–683. 17693054 10.1016/j.janxdis.2007.07.001 PMC2517172

[8] Deshmukh VM, Toelle BG, Usherwood T, et al. The association of comorbid anxiety and depression with asthma-related quality of life and symptom perception in adults. Respirology. 2008;13:695–702. 18513245 10.1111/j.1440-1843.2008.01310.x

[9] Global Initiative for Asthma [Internet] Global Strategy for Asthma Management and Prevention, 2016 [cited 2019 Jan 22]. Available from: www.ginasthma.org.

[10] Yawn B The role of the primary care physician in helping adolescent and adult patients improve asthma control. Mayo Clin Proc. 2011;9:894–902. 10.4065/mcp.2011.0035 PMC3257999 21878602

[11] Howden-Chapman P, Pierse N, Nicholls S, et al. Effects of improved home heating on asthma in community dwelling children: randomised controlled trial. BMJ. 2008;337:1411. 10.1136/bmj.a1411 PMC2658826 18812366

[12] Dietemann A, Bessot JC, Hoyet C, et al. A double-blind, placebo controlled trial of solidified benzyl benzoate applied in dwellings of asthmatic patients sensitive to mites: clinical efficacy and effect on mite allergens. J Allergy Clin Immunol. 1993;91:738–746. 8454796 10.1016/0091-6749(93)90193-j

[13] Sauni R, Uitti J, Jauhiainen M, et al. Remediating buildings damaged by dampness and mould for preventing or reducing respiratory tract symptoms, infections and asthma (Review). Evid Based Child Health. 2013:8;944–1000. 23877912 10.1002/ebch.1914

[14] Takaro TK, Krieger J, Song L, et al. The breathe-easy home: the impact of asthma-friendly home construction on clinical outcomes and trigger exposure. Am J Public Health. 2011;101:55–62. 21148715 10.2105/AJPH.2010.300008 PMC3000722

[15] De Groene GJ, Pal TM, Beach J, et al. Workplace interventions for treatment of occupational asthma: a Cochrane systematic review. Occup Environ Med. 2012;69:373–374. 22267450 10.1136/oemed-2011-100399

[16] Butz AM, Matsui EC, Breysse P, et al. A randomized trial of air cleaners and a health coach to improve indoor air quality for inner-city children with asthma and secondhand smoke exposure. Arch Pediatr Adolesc Med. 2011;165:741–748. 21810636 10.1001/archpediatrics.2011.111 PMC6413330

[17] Lanphear BP, Hornung RW, Khoury J, et al. A. Effects of HEPA air cleaners on unscheduled asthma visits and asthma symptoms for children exposed to secondhand tobacco smoke. Pediatrics. 2011;127:93–101. 21149427 10.1542/peds.2009-2312 PMC3010094

[18] McDonald E, Cook D, Newman T, et al. Effect of air filtration systems on asthma: a systematic review of randomized trials. Chest. 2002;122:1535–1542. 12426250 10.1378/chest.122.5.1535

[19] Warner JA, Frederick JM, Bryant TN, et al. Mechanical ventilation and high-efficiency vacuum cleaning: A combined strategy of mite and mite allergen reduction in the control of mite-sensitive asthma. J Allergy Clin Immunol. 2000;105:75–82. 10629456 10.1016/s0091-6749(00)90181-7

[20] Kilburn SA, Lasserson TJ, McKean MC Pet allergen control measures for allergic asthma in children and adults. Cochrane Database Syst Rev. 2003;1CD002989. 10.1002/14651858.CD002989 PMC8689577 12535446

[21] Singh M, Jaiswal N Dehumidifiers for chronic asthma. Cochrane Database Syst Rev. 2013;6:CD003563. 10.1002/14651858.CD003563.pub2 PMC10646756 23760885

[22] Blackhall K, Appleton S, Cates CJ Ionisers for chronic asthma. Cochrane Database Syst Rev. 2012;9:CD002986. 10.1002/14651858.CD002986.pub2 PMC6483773 22972060

[23] Woodcock A, Forster L, Matthews E, et al. Control of exposure to mite allergen and allergen-impermeable bed covers for adults with asthma. N Engl J Med. 2003;349:225–236. 12867606 10.1056/NEJMoa023175

[24] Glasgow NJ, Ponsonby A-L, Kemp A, et al. Feather bedding and childhood asthma associated with house dust mite sensitisation: a randomised controlled trial. Arch Dis Child. 2011;96:541–547. 21451166 10.1136/adc.2010.189696 PMC3093241

[25] Carter MC, Perzanowski MS, Raymond A, et al. Home intervention in the treatment of asthma among inner-city children. J Allergy Clin Immunol. 2001;108:732–737. 11692097 10.1067/mai.2001.119155

[26] Gøtzsche PC, Hammarquist C, Burr M House dust mite control measures in the management of asthma: meta-analysis. BMJ. 1998;317:1105–1110. 9784442 10.1136/bmj.317.7166.1105 PMC28691

[27] Schatz M, Zeiger RS Ineffectiveness of telephone-based environmental control intervention to improve asthma outcomes. J Allergy Clin Immunol. 2010;126:873–875. 20920777 10.1016/j.jaci.2010.07.035

[28] Kaur B, Rowe BH, Stovold E Vitamin C supplementation for asthma. Cochrane Database Syst Rev. 2013;3:CD000993. 10.1002/14651858.CD000993.pub4 PMC6176495 24155047

[29] Woods RK, Thien FC, Abramson MJ Dietary marine fatty acids (fish oil) for asthma in adults and children. Cochrane Database Syst Rev. 2002;3:CD001283. 10.1002/14651858.CD001283 12137622

[30] Allam MF, Lucane RA Selenium supplementation for asthma. Cochrane Database Syst Rev. 2004;2:CD003538. 10.1002/14651858.CD003538.pub2 PMC9007145 15106206

[31] Pogson Z, McKeever T Dietary sodium manipulation and asthma. Cochrane Database Syst Rev. 2011;3:CD000436. 10.1002/14651858.CD000436.pub3 PMC7032646 21412865

[32] Zhou Y, Yang M, Dong BR Monosodium glutamate avoidance for chronic asthma in adults and children. Cochrane Database Syst Rev. 2012;6:CD004357. 10.1002/14651858.CD004357.pub4 PMC8823518 22696342

[33] Welsh EJ, Bara A, Barley E, et al. Caffeine for asthma. Cochrane Database Syst Rev. 2010;1:CD001112. 10.1002/14651858.CD001112.pub2 PMC7053252 20091514

[34] Chandratilleke MG, Carson KV, Picot J, et al. Physical training for asthma. Cochrane Database Syst Rev. 2012;5:CD001116. 10.1002/14651858.CD001116.pub3 22592674

[35] Beggs S, Foong YC, Le HCT Swimming training for asthma in children and adolescents aged 18 years and under. Cochrane Database Syst Rev. 2013;4:CD009607. 10.1002/14651858.CD009607.pub2 PMC12193991 23633375

[36] Cheng J, Pan T, Ye GH, et al. Calorie controlled diet for chronic asthma. Cochrane Database Syst Rev. 2005;3:CD004674. 10.1002/14651858.CD004674.pub2 PMC12399358 16034941

[37] Milan SJ, Hart A, Wilkinson M Vitamin C for asthma and exercise-induced bronchoconstriction. Cochrane Database Syst Rev. 2014;6 CD010391. 10.1002/14651858.CD010749.pub2 PMC6513032 24936673

[38] Wilkinson M, Hart A, Milan SJ Vitamins C and E for asthma and exercise-induced bronchoconstriction; Cochrane Database Syst Rev. 2014;6 CD010749. 10.1002/14651858.CD010749.pub2 PMC6513032 24936673

[39] Carson KV, Chandratilleke MG, Picot J, et al. Physical training for asthma. Cochrane Database Syst Rev. 2013;9 CD001116. 10.1002/14651858.CD001116.pub4 PMC11930393 24085631

[40] Freitas DA, Holloway EA, Bruno SS, et al. Breathing exercises for adults with asthma. Cochrane Database Syst Rev. 2013;10 CD001277. 10.1002/14651858.CD001277.pub3 24085551

[41] Silva IS, Fregonezi GA, Dias FA, et al. Inspiratory muscle training for asthma. Cochrane Database Syst Rev. 2013;9 CD003792. 10.1002/14651858.CD003792.pub2 PMC7163283 24014205

[42] Grande AJ, Silva V, Andriolo BN, et al. Water-based exercise for adults with asthma. Cochrane Database Syst Rev. 2014;7 CD010456. 10.1002/14651858.CD010456.pub2 PMC11252722 25032820

[43] Adeniyi FB, Young T Weight loss interventions for chronic asthma. Cochrane Database Syst Rev. 2012;7:CD009339. 10.1002/14651858.CD009339.pub2 PMC12075998 22786526

[44] Sheikh A, Alves B, Dhami S Pneumococcal vaccine for asthma. Cochrane Database Syst Rev. 2002;1:CD002165. 10.1002/14651858.CD002165 11869626

[45] Cates CJ, Rowe BH Vaccines for preventing influenza in people with asthma. Cochrane Database Syst Rev. 2013;2:CD000364. 10.1002/14651858.CD000364.pub4 PMC6999427 23450529

[46] McCarney RW, Brinkhaus B, Lasserson TJ, et al. Acupuncture for chronic asthma. Cochrane Database Syst Rev. 2003;3:CD000008. 10.1002/14651858.CD000008.pub2 PMC7061358 14973944

[47] McCarney RW, Linde K, Lasserson TJ Homeopathy for chronic asthma. Cochrane Database Syst Rev. 2004;1:CD000353. 10.1002/14651858.CD000353.pub2 PMC7032670 14973954

[48] Arnold E, Clark CE, Lasserson TJ, et al. Herbal interventions for chronic asthma in adults and children. Cochrane Database Syst Rev. 2008;1:CD005989. 10.1002/14651858.CD005989.pub2 PMC13100575 18254089

[49] Beamon SP, Falkenbach A, Fainburg G, et al. Speleotherapy for asthma. Cochrane Database Syst Rev. 2001;2:CD001741. 10.1002/14651858.CD001741 PMC6435215 10796665

[50] Holloway EA, Ram FS Breathing exercises for asthma. Cochrane Database Syst Rev. 2009;1:CD001277. 10.1002/14651858.CD001277.pub2 14973966

[51] Hondras MA, Linde K, Jones AP Manual therapy for asthma. Cochrane Database Syst Rev. 2005;2:CD001002. 10.1002/14651858.CD001002.pub2 PMC13429289 15846609

[52] Yorke J, Fleming SL, Shuldham C Psychological interventions for adults with asthma. Cochrane Database Syst Rev. 2006;1:CD002982. 10.1002/14651858.CD002982.pub3 PMC7004249 16437449

[53] Yorke J, Fleming SL, Shuldham C Psychological interventions for children with asthma. Cochrane Database Syst Rev. 2005;4:CD003272. 10.1002/14651858.CD003272.pub2 PMC7003566 16235317

[54] Yorke J, Shuldham C Family therapy for asthma in children. Cochrane Database Syst Rev. 2005;2:CD000089. 10.1002/14651858.CD000089.pub2 PMC7038646 15846599

[55] Paudyal P, Hine P, Theadom A, et al. Written emotional disclosure for asthma. Cochrane Database Syst Rev. 2014;5:CD007676. 10.1002/14651858.CD007676.pub2 PMC11254376 24842151

[56] World Health Organization Therapeutic patient education – continuing education programmes for health care providers in the field of prevention of chronic diseases. Geneva: WHO, 1998 [cited 2019 Jan 22]. Available on: http://www.euro.who.int/__data/assets/pdf_le/0007/145294/E63674.pdf.

[57] Boyd M, Lasserson TJ, McKean MC, et al. Interventions for educating children who are at risk of asthma-related emergency department attendance. Cochrane Database Syst Rev. 2009;2:CD001290. 10.1002/14651858.CD001290.pub2 PMC7079713 19370563

[58] Gibson PG, Powell H, Wilson A, et al. Self-management education and regular practitioner review for adults with asthma. Cochrane Database Syst Rev. 2002;3:CD001117. 10.1002/14651858.CD001117 12535399

[59] Wolf F, Guevara JP, Grum CM, et al. Educational interventions for asthma in children. Cochrane Database Syst Rev. 2003;4:CD000326. 10.1002/14651858.CD000326 PMC12668576 12535395

[60] Tapp S, Lasserson TJ, Rowe BH Education interventions for adults who attend the emergency room for acute asthma. Cochrane Database Syst Rev. 2007;3:CD003000. 10.1002/14651858.CD003000.pub2 PMC11491197 17636712

[61] Gibson PG, Powell H, Coughlan J, et al. Limited (information only) patient education programs for adults with asthma. Cochrane Database Syst Rev. 2002;2:CD001005. 10.1002/14651858.CD001005 PMC8407426 10796580

[62] D’Souza W, Burgess C, Ayson M, et al. Trial of a “credit card” asthma self-management plan in a high-risk group of patients with asthma. J Allergy Clin Immunol. 1996;97:1085–1092. 8626986 10.1016/s0091-6749(96)70262-2

[63] Powell H, Gibson PG Options for self-management education for adults with asthma. Cochrane Database Syst Rev. 2003;3:CD004107. 10.1002/14651858.CD004107 PMC8406716 12535511

[64] Bhogal SK, Zemek RL, Ducharme F Written action plans for asthma in children. Cochrane Database Syst Rev. 2006;3:CD005306. 10.1002/14651858.CD005306.pub2 16856090

[65] Peytremann-Bridevaux I, Arditi C, Gex G, et al. Chronic disease management programmes for adults with asthma. Cochrane Database Syst Rev. 2015;5 CD007988. 10.1002/14651858.CD007988.pub2 PMC10640711 26014500

[66] Greineder DK, Loane KC, Parks P Reduction in resource utilization by an asthma outreach program. Arch Pediatr Adolesc Med. 1995;149:415–420. 7704170 10.1001/archpedi.1995.02170160069010

[67] Welsh EJ, Hasan M, Li P. Home-based educational interventions for children with asthma. Cochrane Database Syst Rev. 2011;10:CD008469. 10.1002/14651858.CD008469.pub2 PMC8972064 21975783

[68] Bruzzese JM, Sheares BJ, Vincent EJ, et al. Effects of a school-based intervention for urban adolescents with asthma. A controlled trial. Am J Respir Crit Care Med. 2011;183:998–1006. 21139088 10.1164/rccm.201003-0429OC PMC3086747

[69] Côté J, Bowie DM, Robichaud P Evaluation of two different educational interventions for adult patients consulting with an acute asthma exacerbation. Am J Respir Crit Care Med. 2001;163:1415–1419. 11371411 10.1164/ajrccm.163.6.2006069

[70] Cowie RL, Revitt SG, Underwood MF, et al. The effect of a peak flow-based action plan in the prevention of exacerbations of asthma. Chest. 1997;112:1534–1538. 9404750 10.1378/chest.112.6.1534

[71] Coté J, Cartier A, Robichaud P, et al. Influence on asthma morbidity of asthma education programs based on self-management plans following treatment optimization. Am J Respir Crit Care Med. 1997;155:1509–1514. 9154850 10.1164/ajrccm.155.5.9154850

[72] de Jongh T, Gurol-Urganci I, Vodopivec-Jamsek V, et l. Mobile phone messaging for facilitating self-management of long-term illnesses. Cochrane Database Syst Rev. 2012;12 CD007459. 23235644 10.1002/14651858.CD007459.pub2 PMC6486189

[73] Runge C, Lecheler J, Horn M, et al. Outcomes of a web-based patient education program for asthmatic children and adolescents. Chest. 2006;129:581–593. 16537855 10.1378/chest.129.3.581

[74] Apter AJ, Wang X, Bogen DK, et al. Problem solving to improve adherence and asthma outcomes in urban adults with moderate or severe asthma: a randomized controlled trial. J Allergy Clin Immunol. 2011;128:516–523. 21704360 10.1016/j.jaci.2011.05.010 PMC3164914

[75] Chang AB, Taylor B, Masters IB, et al. Indigenous healthcare worker involvement for Indigenous adults and children with asthma. Cochrane Database Syst Rev. 2010;5:CD006344. 10.1002/14651858.CD006344.pub3 PMC13139708 20464742

[76] Bailey EJ, Cates CJ, Kruske SG, et al. Culture-specific programs for children and adults from minority groups who have asthma. Cochrane Database Syst Rev. 2009;2:CD006580. 10.1002/14651858.CD006580.pub2 18425956

[77] Canino G, Vila D, Normand S-LT, et al. Reducing asthma health disparities in poor Puerto Rican children: the effectiveness of a culturally tailored family intervention. J Allergy Clin Immunol. 2008;121:665–670. 18061648 10.1016/j.jaci.2007.10.022 PMC3136215

[78] Marcano Belisario JS, Huckvale K, Greenfield G, et al. Smartphone and tablet self management apps for asthma. Cochrane Database Syst Rev. 2013;11 CD010013. 10.1002/14651858.CD010013.pub2 PMC6486323 24282112

[79] Baishnab E, Karner C Primary care based clinics for asthma. Cochrane Database Syst Rev. 2012;4:CD003533. 10.1002/14651858.CD003533.pub2 PMC7154586 22513914

[80] Kuethe MC, Vaessen-Verberne AAPH, Elbers RG, et al. Nurse versus physician-led care for the management of asthma. Cochrane Database Syst Rev. 2013;2:CD009296. 10.1002/14651858.CD009296.pub2 PMC11810130 23450599

[81] Pande S, Hiller JE, Nkansah N, et al. The effect of pharmacist-provided non-dispensing services on patient outcomes, health service utilisation and costs in low- and middle-income countries. Cochrane Database Syst Rev. 2013;2:CD010398. 10.1002/14651858.CD010398 PMC9829534 23450614

[82] McLean S, Chandler D, Nurmatov U, et al. Telehealthcare for asthma: a Cochrane review. CMAJ. 2011;183:E733–742. 21746825 10.1503/cmaj.101146 PMC3153544

[83] Halterman JS, Fagnano M, Montes G, et al. The school-based preventive asthma care trial: results of a pilot study. J Pediatr. 2012;161:1109–1115. 22785264 10.1016/j.jpeds.2012.05.059 PMC3470823

[84] Zorc JJ, Chew A, Allen JL, et al. Beliefs and barriers to follow-up after an emergency department asthma visit: a randomized trial. Pediatrics. 2009;124:1135–1142. 19786448 10.1542/peds.2008-3352 PMC2803082

[85] Evans R, Gergen PJ, Mitchell H, et al. A randomized clinical trial to reduce asthma morbidity among inner-city children: results of the National Cooperative Inner-City Asthma Study. J Pediatr. 1999;135:332–338. 10484799 10.1016/s0022-3476(99)70130-7

[86] Morgan WJ, Crain EF, Gruchalla RS, et al. Results of a home- based environmental intervention among urban children with asthma. N Engl J Med. 2004;351:1068–1080. 15356304 10.1056/NEJMoa032097

[87] Teach SJ, Crain EF, Quint DM, et al. Improved asthma outcomes in a high-morbidity pediatric population: results of an emergency department-based randomized clinical trial. Arch Pediatr Adolesc Med. 2006;160:535–541. 16651498 10.1001/archpedi.160.5.535

[88] Kintner EK, Sikorskii A Randomized clinical trial of a school-based academic and counseling program for older school-age students. Nurs Res. 2009;58:321–331. 19752672 10.1097/NNR.0b013e3181b4b60e PMC2884155

[89] Halterman JS, Fagnano M, Tremblay PJ, et al. Prompting asthma intervention in Rochester—uniting parents and providers (PAIR-UP): a randomized trial. JAMA Pediatr. 2014;168:e141983. 25288141 10.1001/jamapediatrics.2014.1983 PMC4232370

[90] Halterman JS, Szilagyi PG, Fisher SG, et al. Randomized controlled trial to improve care for urban children with asthma: results of the School-Based Asthma Therapy trial. Arch Pediatr Adolesc Med. 2011;165:262–268. 21383275 10.1001/archpediatrics.2011.1 PMC3600609

[91] Klinnert MD, Liu AH, Pearson MR, et al. Outcome of a randomized multifaceted intervention with low-income families of wheezing infants. Arch Pediatr Adolesc Med. 2007;161:783–190. 15630062 10.1001/archpedi.159.1.75

[92] Klinnert MD, Liu AH, Pearson MR, et al. Short-term impact of a randomized multifaceted intervention for wheezing infants in low-income families. Arch Pediatr Adolesc Med. 2005;159:75–82. 15630062 10.1001/archpedi.159.1.75

[93] Fisher EB, Strunk RC, Highstein GR, et al. A randomized control-led evaluation of the effect of community health workers on hospitalization for asthma: the asthma coach. Arch Pediatr Adolesc Med. 2009;163:225–232. 19255389 10.1001/archpediatrics.2008.577

[94] Krieger JW, Takaro TK, Song L, et al. The Seattle-King County Healthy Homes Project: a randomized, controlled trial of a community health worker intervention to decrease exposure to indoor asthma triggers. Am J Public Health. 2005;95:652–659. 15798126 10.2105/AJPH.2004.042994 PMC1449237

[95] Allen J, Gay B, Crebolder H, et al. The European definitions of the key features of the discipline of general practice: the role of the GP and core competencies. Br J Gen Pract. 2002;52:526–527. 12051237 PMC1314348

[96] Gold D, Adamkiewicz G, Arshad SH, et al. NIAID, NIEHS, NHLBI, MCAN Workshop Report: the indoor environment and childhood asthma: implications for home environmental intervention in asthma prevention and management. J Allergy Clinical Immunol. 2017;140:933–949. 28502823 10.1016/j.jaci.2017.04.024 PMC5632590

[97] Jackson DJ, Sykes A, Mallia P, Johnston SL Asthma exacerbations: origin, effect, and prevention. J Allergy Clinical Immunol. 2011;128:1165–1174. 22133317 10.1016/j.jaci.2011.10.024 PMC7172902

[98] Busse WW, Lemanske RF, Gern JE The role of viral respiratory infections in asthma and asthma exacerbations. Lancet. 2010;376:826–834. 20816549 10.1016/S0140-6736(10)61380-3 PMC2972660

[99] To T1, C Daly, R Feldman, et al. Results from a community-based program evaluating the effect of changing smoking status on asthma symptom control. BMC Public Health. 2012;12:293. 22520046 10.1186/1471-2458-12-293 PMC3464678

[100] Borys JM, Le Bodo Y, Jebb SA, et al. EPODE approach for childhood obesity prevention: methods, progress and international development: EPODE approach for obesity prevention. Obesity Reviews. 2012;13:299–315. 22106871 10.1111/j.1467-789X.2011.00950.x PMC3492853

[101] Borys JM, Valdeyron L, Levy E, et al. EPODE – a model for reducing the incidence of obesity and weight-related comorbidities. Eur Endocrinol. 2013;9:116–20. 29922365 10.17925/EE.2013.09.02.116 PMC6003578

[102] Borys JM, Richard P, Ruault du Plessis H, et al. Tackling health inequities and reducing obesity prevalence: the EPODE community-based approach. Ann Nutr Metab. 2016;68(suppl 2):35–38. 10.1159/000446223 27300305

[103] Wagner E Chronic disease management: what will it take to improve care for chronic illness? Eff Clin Pract. 1998;1:2–4. 10345255

[104] Halterman JS, Borrelli B, Fisher S, et al. Improving care for urban children with asthma: design and methods of the School-Based Asthma Therapy (SBAT) Trial. J Asthma. 2008;45:279–286. 18446591 10.1080/02770900701854908 PMC2605580

[105] Kennedy S, Bailey R, Jaffee K, et al. Effectiveness of evidence-based asthma interventions. Pediatrics. 2017;139(6):e20164221. 10.1542/peds.2016-4221 28562279

